# Cardiac Myxomas As Chameleons: A Scoping Review of Their Paraneoplastic Presentations

**DOI:** 10.7759/cureus.37558

**Published:** 2023-04-14

**Authors:** Tomas Escobar Gil, Alejandro Echavarria Cross, Sofía Valencia Barrera, Antonia Bustamante Omaña

**Affiliations:** 1 Internal Medicine, The University of New Mexico School of Medicine, Albuquerque, USA; 2 Internal Medicine, Universidad CES, Medellín, COL

**Keywords:** echocardiography in cardio-oncology, heart surgery, adult cardiac surgery, embolus, benign tumors, right atrial myxoma, paraneoplastic syndromes, oncology, cardiology, tumor

## Abstract

This scoping review aims to explore the relationship between cardiac myxomas (CMs) and paraneoplastic syndromes (PS). CMs are the most common tumors in the heart, with the majority located in the left atrium, and are often associated with a triad of obstructive, embolic, and constitutional symptoms. However, they can also present with unrelated symptoms that are part of a PS. This study performed a thorough literature search of 11 databases and included 12 papers in the final review. All of the patients were diagnosed with atrial myxoma, which initially presented as a PS. Surgery was the curative measure in all cases and resulted in remission in every case, with patients reporting resolution of symptoms at follow-up. The majority of patients in the study were female, with comorbid rheumatologic conditions often present. This study highlights the heterogeneity of presentations of CMs and their associated PS.

## Introduction and background

Cardiac myxomas (CMs) are the most common tumors in the heart, with a prevalence of seven cases per 10,000 population [1,2]. They are intracavitary tumors, which are usually round or oval-shaped, pedunculated, and mobile [1]. The vast majority are located in the left atrium (75%), followed by the right atrium (20%), and just a minority are rarely found in the ventricles [1,3].

Although CMs can sometimes be asymptomatic and diagnosed incidentally, they usually present with a wide range of symptoms. The classical triad of obstructive, embolic, and constitutional symptoms is commonly associated with CMs [3]. However, patients may present with symptoms that may not seem related to the tumor at all, such as bone pain, rashes, sensory deficits, seizures, arthritis, or even exacerbations of uncontrolled asthma [4]. These unrelated symptoms are what is called paraneoplastic syndrome (PS), a set of signs and symptoms that are the consequence of the production of chemical signaling molecules (such as hormones or cytokines) by tumor cells or by an immune response against a tumor [4,5].

Before the advent of cardiac imaging techniques, CMs were diagnosed postmortem. In 1952, the first premortem diagnosis was made with angiography. Later, in 1968, the first diagnosis of left atrial myxoma was made with echocardiography using motion mode (M-mode). With the increasing use of echocardiography and point-of-care ultrasound (POCUS), most cases of myxoma are diagnosed early in their course [6].

Interestingly, and according to some authors, in some patients with myxomas and a paraneoplastic initial presentation, the unrelated symptom seems to resolve as soon as the tumor is surgically resected [4]. These authors have suggested that a potential association between cardiac myxomas and paraneoplastic syndromes exists and that surgical resection may effectively treat both the tumor and associated symptoms [4]. However, further research is needed to establish a causal relationship between tumor resection and symptom resolution in these cases.

Our study aims to search the literature and discover how heterogeneous these paraneoplastic presentations have been in the past to contribute to the understanding of this tumor's behavior, manifestations, and presentation.

## Review

Materials and methods

**Figure 1:**
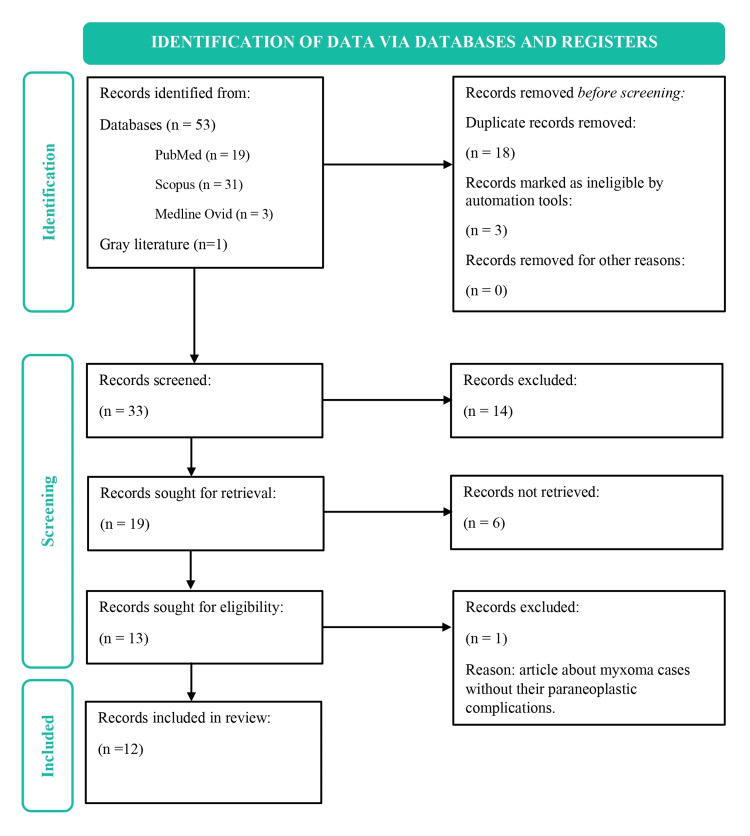
Flowchart for article selection

To conduct this review, the critical appraisal skills programme (CASP/CASPe) guidelines were utilized as a model, while the preferred reporting items for systematic reviews and meta-analyses (PRISMA) statement was followed for designing the flowchart (Figure 1). A thorough literature search was performed across 11 databases, namely PubMed, Cochrane Library, Latin American & Caribbean Health Sciences Literature (LILACS), Ovid Medical Literature Analysis and Retrieval System Online (MEDLINE), The Journal of the American Medical Association (JAMA) Network, IBECS, Cuba Medicina (CUMED), Scopus, Scientific Electronic Library Online (SciELO), MEDLINE-EBSCO, and Taylor & Francis Online, using a combination of MeSH (Medical Subject Heading) and DeCS (Descriptores en Ciencias de la Salud) terms. Grey literature sources (Google Scholar and Open Grey) were also included in our review.

The terms “myxoma”, “paraneoplastic”, and “paraneoplastic syndrome”, were used in databases in English, while the terms “mixoma”, “síndrome paraneoplásico”, and “paraneoplásico” were used in databases in Spanish. Additionally, Boolean operators “AND” and “Y” were used. Only articles in English or Spanish were considered for this review, which ranges from 1995 to 2023. 

The study types and inclusion criteria were carefully chosen to select only those with original data that evaluated the relationship between myxomas and their presentation as paraneoplastic syndromes in human subjects. Only specific study types were included, such as case reports, case series, letters to editors depicting cases, cross-sectional studies, retrospective or prospective cohort studies, case-control studies, and randomized clinical trials. Systematic review articles, mini-review articles, meta-analysis articles, opinion articles, and complete books were excluded from the analysis.

Among the exclusion criteria were the following: studies that focused solely on myxomas without the paraneoplastic component; studies about malignant neoplasms of the heart or noncardiac primary tumors; and studies about noncardiac myxomas. Only articles that met all of the inclusion criteria were considered for full-text evaluation. Details of this process can be found in Figure 1.

Results

After an extensive search of 11 databases and additional grey literature sources, 53 records were obtained. Through careful screening and evaluation, a final selection of 12 papers was identified for the scoping review, including 11 case reports and one case series.

All of the cases identified had a diagnosis of atrial myxoma that initially presented as a PS. The demographic data of patients were included, as well as their comorbidities. Pathology reports and detailed tumor size descriptions were provided for every case, with all cases consisting of myxomatous tumors with diameters ranging from 1.7cm in one case to a maximum of 10cm in one of the cases. Surgery was found to be the curative measure in all cases, and the outcome of each case was further described. These findings can be found in Figure 1.

The majority of patients in the study were female, with males comprising only 14% of cases. Additionally, the median age of patients was 37.2 years old. Some of the comorbid presentations included rheumatologic manifestations like positive autoantibodies in one patient, Raynaud's phenomenon in one patient, arthritis in one patient, and polyarthritis in another; anemia in two of the patients; hypertension in two of the patients; herpes zoster and panic disorder in one of the patients; vertigo in one of the patients; and developmental delay in one of the patients. Four patients had no known comorbidities. The data presented in Table 1 reflects that there is indeed an association between atrial myxomas and paraneoplastic syndromes; however, causality cannot be established through clinical case reports. The presence of CM could be caused by the syndrome itself or could simply be a coincidental finding, adding another confounding factor.

**Table 1:** Case reports and case series depicting cardiac myxomas that initially presented as a PS CT: computed tomography; SLE: systemic lupus erythematosus; ESR: erythrocyte sedimentation rate; PS: paraneoplastic syndrome

AUTHOR	TYPE OF STUDY	AGE OF PATIENT	GENDER	DIAGNOSIS	DIMENSIONS	COMORBIDITIES	TYPE OF PS	TREATMENT	OUTCOME
Chikkabyrappa [7]	Case report	8	Female	Pathology confirmed the diagnosis of myxoma	1.7 × 1.8cm (echocardiography report)	Mild developmental delay	Chorea	Surgical intervention	Resolution of chorea soon after the removal of myxoma
Cobo [8]	Case report	33	Female	Pathology confirmed the diagnosis of myxoma	9 × 3cm (echocardiography report)	No comorbidities only use of oral contraceptives	Bilateral pulmonary embolism	Surgical intervention	Resolution of pulmonary embolism
Durand [9]	Case report	20	Female	Myxoma, with areas of stellate-shaped and fusiform cells on a myxoid and fibrous background, and necrotized eosinophilic areas	3 × 2cm (echocardiography report)	No history of thrombosis or SLE criteria but positive antibodies: anti-nuclear, lupus anticoagulant, antiphospholipid in low titer	Immune thrombocytopenia	Surgical intervention	Resolution of thrombocytopenia
Fatimi [10]	Case report	64	Male	Myxomatous tissue, where neoplastic cells were arranged in the form of cords	10 × 4 × 6cm	Hypertension and ischemic heart disease	Acquired severe factor VII (FVII) deficiency	Surgical intervention	Normalization of factor VII levels
Macias [11]	Case report	13	Female	Pathology confirmed the diagnosis of myxoma	3.5 × 3.5 × 2.5cm	No comorbidities	Vasculitis	Surgical intervention	Complete resolution of symptoms at eight-month follow-up.
Misago [12]	Case report	35	Female	Small number of spindle-shaped in small nests or solitary units, within the eosinophilic, amorphous stroma with partial deposition of a myxoid substance	7 × 4 × 4cm	Iron deficiency anemia	Erythematous papules	Surgical intervention	Unknown
Momtahen [13]	Case report	21	Female	Myxoma cells surrounding dilated vessels and scattered in a myxoid matrix	4.3 × 3cm (echocardiography report)	Hypertension, vertigo, and an incidental finding of thickened calvarium on CT done for vertigo workup	Increased diffuse bone activity in right scapula, left femur, and calvarium on bone scan	Surgical intervention	Resolution of the increased bone activity
Nishizaki [14]	Case report	40	Female	Mass strongly suspicious of a left atrial myxoma, pathology not reported	2.4 × 1.4cm (echocardiography report)	Herpes zoster, panic disorder	Esophageal ulcer	Surgical intervention	No recurrence of thoracic or epigastric pain for three months
Padhan [15]	Case report	65	Female	Myxoid areas and scanty cellularity, spindle, and stellate cells arranged around thin-walled blood vessels	Approximately 0.8 × 1cm (echocardiography report)	Anemia	Polyarthritis	Surgical intervention	Resolution of systemic symptoms, including arthritis with a reduction in ESR
Santangeli [16]	Case report	51	Male	Groups of scattered stellate, spindle, and round cells in a strand of amorphous eosinophilic material with areas of focal hemorrhage,	Largest diameter of 5cm	No comorbidities	Sensory neuropathy	Surgical intervention	Resolution of neurological symptoms and nerve conduction study normalization.
Suh [17]	Case report	68	Female	Pathology confirmed the diagnosis of myxoma	2.9 × 2.5cm (echocardiography report)	Arthritis	Polyneuropathy	Surgical intervention	Marked improvement of nerve conduction studies
Zamora [18 ]	Case series	32	Female	Myxoma-type mesenchymal neoplasm with cells with small ovoid and spindle-shaped nuclei containing stellate-edged eosinophilic cytoplasm overlaying a myxoid-appearing stroma	4.5 × 3 × 2cm	No comorbidities	Neurologic constellation of seizures, hemiparesis, peripheral cyanosis, and syncope	Surgical intervention	Unknown
Zamora	Case series	32	Female	Benign neoplastic lesion consisting of a stroma hypocellular myxoid with extensive interstitial hemorrhage, the proliferation of capillaries with the presence of elongated and small stellate cells without atypia, compatible with a myxoma-type tumor	13cm² (echocardiography report)	Raynaud phenomenon	Cyanosis, hemoptysis	Surgical intervention	Resolution of symptoms

At surgery follow-up, all but one of the patients reported resolution of symptoms and no further signs of paraneoplastic disease (the remaining case did not report any follow-up data), indicating the success of the surgical intervention.

Discussion

CMs are unique medical entities. Although they are benign, they can cause a wide array of symptoms and complications. They can masquerade as other entities and present in a paraneoplastic manner. As the understanding of this disease continues to evolve, we conducted this scoping review to identify recent cases in which myxomas presented as PS. The findings can be visualized in Table 1.

Just like Smith et al. in their review of the literature [4], we found a sample of patients who had a myxoma diagnosis that initially presented as a PS [7-18]. Some articles suggest grouping PS by the nature of the symptoms they experience, whether they are arthritic, hematologic, neurologic, respiratory, gastrointestinal, or dermatologic. The most common events include constitutional symptoms at 45-50%, hematological effects at 20-25%, and vasculitis/vasculopathy effects at 12% of cases, the latter even describing embolic events as being part of a "blue digit syndrome" [4,19,20]. Some uncommon manifestations associated with CMs include asthma, chorea, acute pancreatitis, iridocyclitis, worsening of systemic lupus erythematosus (SLE), and antiphospholipid antibodies [4]. One of the patients in the studies we found presented had esophageal ulcers and another had a bilateral pulmonary embolism (in the context of left atrial myxoma). The latter is not a common presentation of PS in patients with myxomas.

The relevance of all these reports relies on the fact that these manifestations resolved with myxoma excision, suggesting a relationship with the myxoma or indicating a possible association with it [4,7]. Limitations of our study include the fact that only articles in English and Spanish were included, that PS in the context of CM might be underreported, and that the sample of our review might not be representative of the true population.

CMs have been found to exhibit several cytokine abnormalities that can cause recurrence, local and systemic invasion, and paraneoplastic manifestations. Elevated levels of interleukin-6 (IL-6), vascular endothelial growth factor (VEGF), basic fibroblast growth factor (bFGF), and monocyte chemotactic protein-1 (MCP-1) have been implicated in the pathogenesis of myxomas. Excessive IL-6 production is associated with local myxoma growth, and frequent recurrence, and literature reports a relationship between distant myxoma metastases and PS. VEGF expression was found to be high in all myxomas analyzed, and it is inversely proportional to tumor size and correlated to the microvessel density of the tumor. The bFGF and the FGFR receptors have been shown to have increased expression in the majority of myxomas, and increased microvessel density occurred in myxomas with high bFGF or FGFR-1 expression. MCP-1 and TP also contribute to the pathogenesis or recurrence of myxomas by inducing angiogenesis. Several cytokine abnormalities have been identified, and no studies have been published on anti-cytokine therapies directed at the humoral factors produced by myxomas [4,21].

In the cases we documented, myxomas had an average diameter of 5.76cm. As advancements in imaging techniques continue to evolve, surgical methods to establish a conclusive diagnosis will soon be outdated. When discussing a cardiac mass, the clinical context is crucial in identifying the etiology of the cardiac mass or lesion. The etiology of a tumor is determined by four factors: patient age, epidemiologic likelihood, tumor location, and noninvasive tissue characterization by cardiovascular magnetic resonance imaging (CMR) [2].

Multimodality imaging is crucial in identifying the presence of a cardiac tumor and its location within the heart structures. Two-dimensional transthoracic echocardiography (TTE) is the primary imaging modality, while transesophageal echocardiography (TEE) is commonly used when a valvular lesion is suspected. CMR offers a comprehensive evaluation of the mass, including its potential involvement with the cardiac chambers and pericardium, and provides information about the extracardiac structures and surrounding anatomy, making it useful in surgical planning. Characteristics that can be evaluated by CMR include morphology, dimensions, location, extension, homogeneity, and the presence of infiltration in surrounding tissues. Although pathological evidence remains the gold standard for diagnosis, imaging techniques are commonly used in clinical practice [2,3,6,22,23].

## Conclusions

Overall, this text highlights the constellation of clinical manifestations related to CMs. The diversity of presentations and outcomes observed in the cases we found in our literature search also underscores the need for personalized approaches to managing patients with myxomas, considering their individual characteristics and comorbidities. CMs can present with the classical triad of obstructive, embolic, and constitutional symptoms, but they can also present with unrelated symptoms that are part of a PS.

In our search, the vast majority of patients were female with underlying rheumatologic conditions, making it difficult to differentiate between whether it was a real PS or whether it was caused by their underlying rheumatic condition. Also contributing to this was the fact that when surgical management was performed, despite the persistent underlying disease, the symptoms disappeared. Further research is needed to establish a causal relationship between tumor resection and symptom resolution in these cases. Future studies could further delve into the molecular mechanisms and genetic factors underlying the development and progression of myxomas, as well as the potential use of advanced imaging techniques and targeted therapies to improve the diagnosis and management of these tumors. Surgical removal is a potential procedure to take into account when no other treatment has led to a resolution of symptoms.
